# Framing effects on physical effort avoidance in younger versus older adults

**DOI:** 10.1002/alz70857_105261

**Published:** 2025-12-25

**Authors:** Krithika Gilari, Sanjana Mamathasateesh, Sri Kari, Nithish Oommen, Robin C. Hilsabeck, Bonnie M Scott

**Affiliations:** ^1^ The University of Texas at Austin Dell Medical School, Austin, TX, USA; ^2^ UT Health San Antonio, San Antonio, TX, USA

## Abstract

**Background:**

(1) Analyze the effects of age [Younger (YA) vs. Older (OA) Adults] and framing (Risk vs. Reward) on effort‐based decision‐making using a modified version of the Effort Expenditure for Rewards Task (M‐EEfRT), in which participants can choose to exert greater physical effort (i.e., finger tapping) to either gain or avoid losing rewards, and (2) Investigate the relationship between M‐EEfRT performance parameters and self‐reported symptoms of mood and motivation. Based on previous research, we hypothesized that (1) YAs would expend more effort [DV=Hard Task Selection Frequency (HTSF)] to gain rewards, while OAs would expend more effort to prevent losing rewards already acquired, and (2) effort expenditure on both trial types would be more strongly related to self‐reported symptoms of motivation than mood.

**Method:**

A cognitively normal sample of 52 YAs (ages 18‐30) and 45 OAs (ages 60‐80) completed the M‐EEfRT and self‐report questionnaires assessing mood (PANAS: Positive and Negative Affect Schedule) and motivation (DAS: Dimensional Apathy Scale).

**Result:**

Overall, there were no significant between‐group differences in effort expenditure (HTSF: YA=56%, OA=61%, Eta^2^ = 0.01). On risk trials, YAs had significantly faster decision reaction times (DRT) and lower effort expenditure (HTSF: YA=34%, OA=55%, Eta^2^ = 0.07) than OAs despite no significant differences in successful trial completion (STC). On reward trials, YAs had significantly higher STC (YA=89%, OA=77%, Eta^2^ = 0.05) and faster DRT than OAs, while only a statistical trend (*p* = 0.06) was found for greater effort expenditure in YA vs. OA groups (HTSF: YA=78%, OA=66%, Eta^2^ = 0.04). Although DRT was correlated with mood, effort expenditure was not significantly associated with either self‐report questionnaire.

**Conclusion:**

The results of this study were partially consistent with our hypotheses, as the effect of framing on effort expenditure varied by age group. However, performance parameters were not significantly correlated with self‐report questionnaires. Given that a similar lack of correspondence between subjective self‐report and objective behavioral performance is often found in the literature, we plan to explore alternative methods of task validation (e.g., activity trackers, sociodemographic equivalence, etc.) in our future work.

